# Penetrating Neck Injury: Double Jeopardy of a Complex Aerodigestive Dilemma

**DOI:** 10.7759/cureus.39533

**Published:** 2023-05-26

**Authors:** Khaled Aljohani, Ahad Alsaud, Fayez G Aldarsouni, Hosam Alruwaite, Norah M Alsubaie

**Affiliations:** 1 Department of Surgery, King Saud University Medical City, Riyadh, SAU; 2 Department of Emergency Medicine, College of Medicine, King Saud University Medical City, Riyadh, SAU

**Keywords:** airway management, drug addiction, ent surgery, surgical case reports, emergency & acute care, penetrating trauma neck, emergency surgical airway, acute care surgery and trauma, penetrating stab wound, major trauma

## Abstract

The neck is a critical region containing many essential structures. Before surgical intervention, it is crucial to assess the adequacy of the airway and circulation, as well as the presence of any skeletal or neurological damage. Here, we present a case of a 33-year-old male with a background of amphetamine abuse who presented to our emergency department with a penetrating neck injury just below the mandible at the hypopharynx level, resulting in an upper zone II neck injury with complete separation of the airway. The patient was taken immediately to the operating room for exploration. Airways were managed by direct intubation, hemostasis was maintained, and the open laryngeal injury was repaired. After the surgery, this patient was transferred to the intensive care unit for two days and discharged after a satisfactory full recovery. Penetrating neck injuries are rare but often fatal. Advanced trauma life support guidelines emphasize the importance of managing the airway as the first action. Providing multidisciplinary care before, during, and after trauma can help prevent and treat such incidents.

## Introduction

Penetrating neck injury (PNI), characterized by a disruption of the platysma muscle, is associated with a mortality rate of up to 10% in the United States [1]. This is attributed to the viability of this area because it contains many important vascular, respiratory, digestive, and neurological structures [2,3]. Optimizing management demands timely and effective intervention, adequate assessment of the airways and circulation, and checking for the presence of any skeletal or neurological damage. There is still a need for a concise statement regarding the management of PNI [3]. We report a case of a 33-year-old male with a background of amphetamine-induced psychosis who presented to the emergency department (ED) with a severe neck impact that caused a complete transection of the hypopharyngeal structures. We also discuss relevant airway management and review the current literature.

## Case presentation

**Figure 1:**
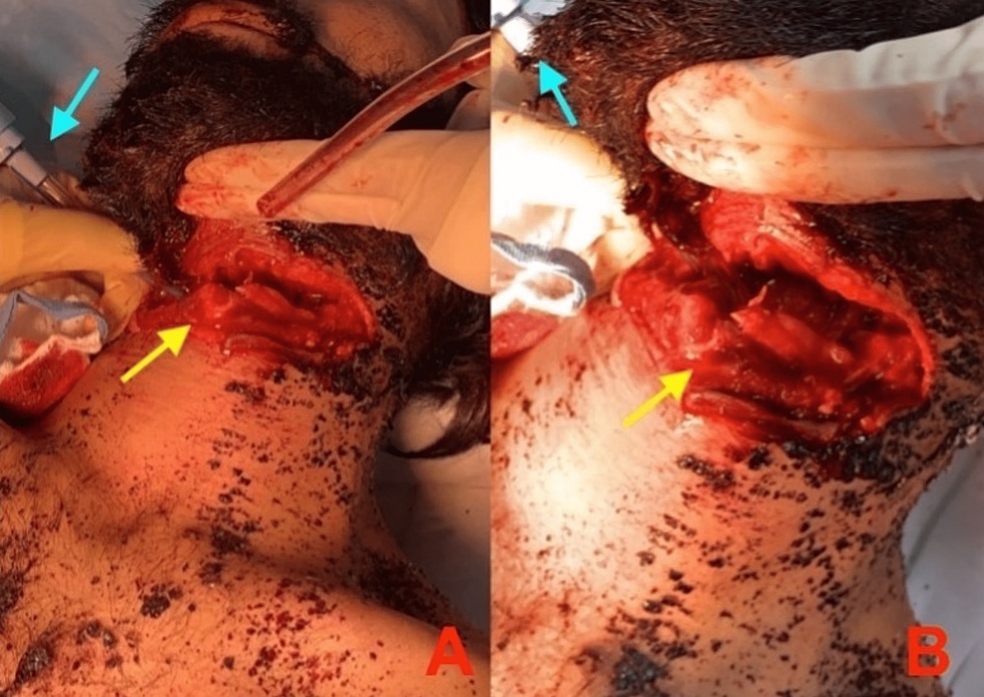
Lateral view of the neck laceration. Providing a temporary airway (ET) tube is essential until a definitive airway can be established. Careful exposure of the entire bony and cartilaginous structures is necessary due to the possibility that certain fractures may not be readily visible. (A) The injury appeared deeper on the left side compared to the right. (B) Distortion of the anatomy. Blue arrow denotes the endotracheal tube. Yellow arrow denotes thyroid cartilage.

A 33-year-old man with amphetamine-induced psychosis presented to the emergency department (ED) with a neck injury caused by a large knife just below the mandible at the level of the hypopharynx. The patient was hemodynamically stable upon arrival and conscious, with a Glasgow coma scale score of 13 out of 15. While on room air, he was breathing spontaneously and had equal air entry bilaterally, with no esophageal injury or neurological abnormalities. On examination, the airways were partially exposed due to a transverse cut wound at upper zone two extending between the right and left sternocleidomastoid muscles. We later discovered that this injury was associated with a complete separation of the airway superior to the thyroid cartilage and bleeding, which we controlled on applying direct pressure. The patient was then taken to the operating room (OR), where the trauma team initially managed the airway with an endotracheal tube size of 7 millimeters inserted into the trachea via the wound (Figures 1A, 1B).

**Figure 2:**
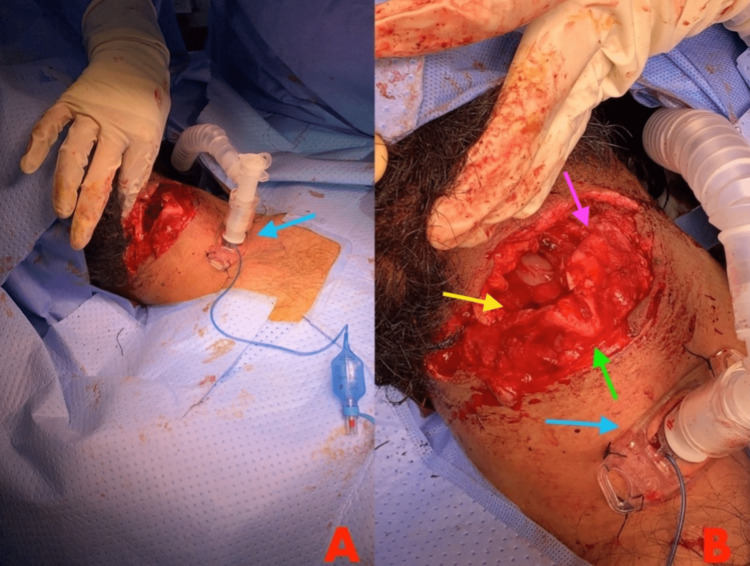
Intraoperative distal view of the neck wound highlights tracheostomy positioning on the neck. The patient's tracheostomy was applied through a separate incision site away from the primary injury site. (A) Distal view of the injury. (B) Proximal view of the injury. Yellow arrow denotes transacted right thyroidal alar cartilage. Pink arrow denotes fractured left thyroidal alar cartilage. Blue arrow denotes the tracheostomy tube. Green arrow denotes the laryngeal prominence of the thyroid cartilage.

The patient was shifted to the OR table for a formal tracheostomy through a separate incision site away from the primary injury site (Figures 2A, 2B). Avoiding inter-planar violation, the patient's airway was connected to the ventilator following the successful tracheostomy.

An intraoperative laryngoscope and endoscope examination were carried out prior to the patient's neck exploration in the operating room. The neck of this patient was completely transected. The transection involved the thyroid cartilage, hyoid bone, aryepiglottic fold, and epiglottis. This was associated with a full-thickness cut in the right upper part of the thyroid lamina, which was displaced and attached only posteriorly, and the left anterior upper part of the thyroid lamina, which was stable. The wound was further explored. The wound was more superficial on the right side and deeper on the left. Upon further exploration of the wound, it was found to be associated with a laceration that separated the epiglottis and vocal cords at the level of the hypopharynx, as well as a fracture of the right thyroid and thyroid alar cartilage (Figure 2B).

Upon assessment of the wound vasculature, active bleeding was discovered from the left facial vein and the anterior jugular veins. The bleeding was thoroughly controlled, and the facial vein transection was managed by ligation. Additionally, a complete transection of the bilateral anterior jugular veins was identified and subsequently managed by meticulous proximal and distal ligation to ensure hemostasis. The internal jugular and the vagus nerve were identified, and the left carotid sheet was dissected, revealing no evidence of injury. After the neck wound closure, this patient's examination was concluded with an esophagogastroduodenoscopy to evaluate the cervical esophagus; the scope was successfully passed through the oropharynx and area of repair down to the esophagus. No hematomas were found distal to the injury on the endoscopic examination. A nasogastric tube (NGT) was inserted under direct vision afterward. The airway was secured with a tracheostomy.

**Figure 3:**
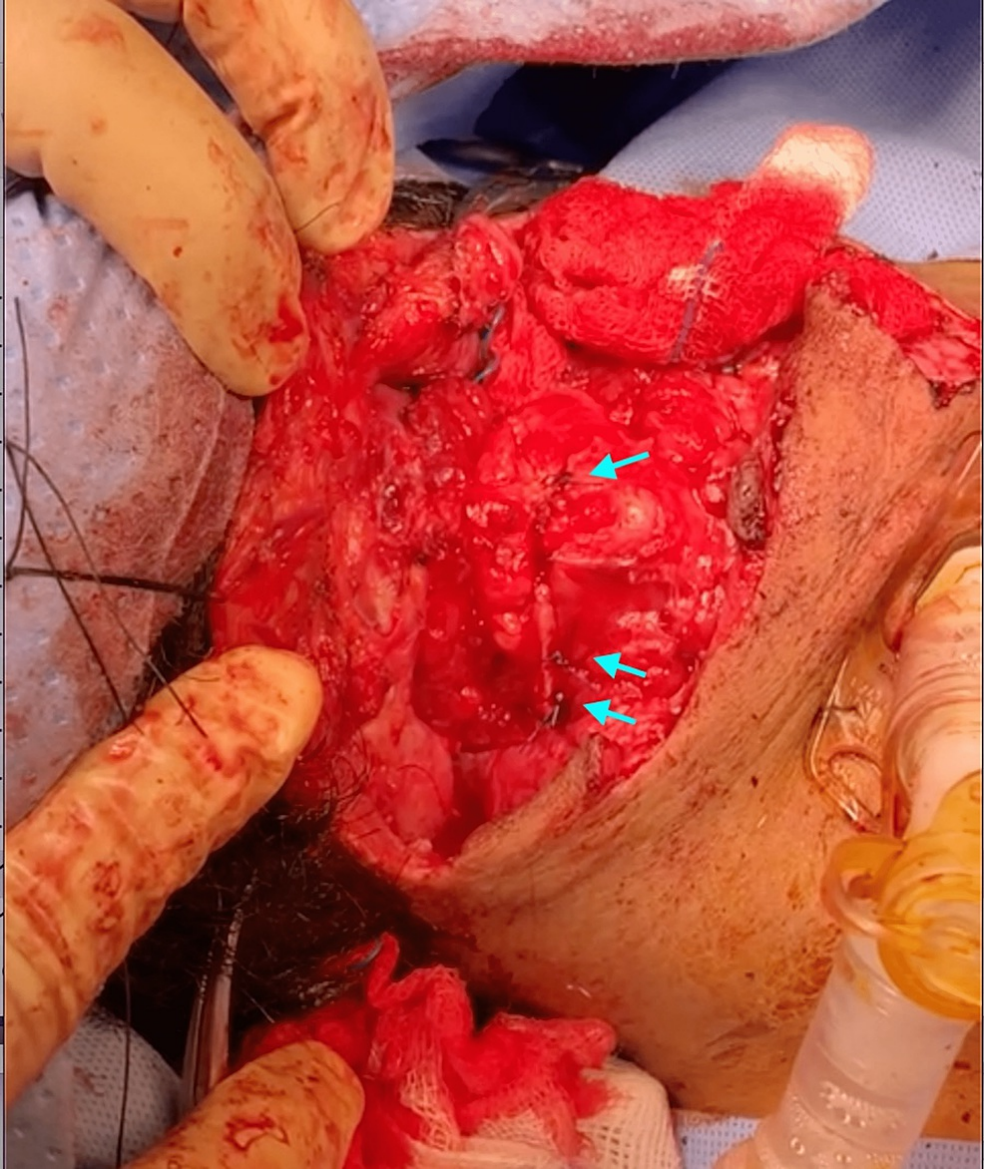
Intraoperative view of the explored neck wound. The epiglottis base was sutured back to the laryngeal surface of the thyroid lamina in the midline by three stitches. Blue arrows denote stitches.

The ear, nose, and throat team repaired the hypopharynx; the right and left thyroid lamina fracture lines were sutured bilaterally by polypropylene 2.0 sutures (Ethicon Company, New Jersey, USA). The patient's upper part of the chest and chin were fixed using micro-suture 3.0 (Unimed Company, Lausanne, Switzerland) to keep the neck at complete flexion at all times. The epiglottis base was sutured back to the laryngeal surface of the thyroid lamina in the midline by three interrupted stitches using polypropylene 2.0 (Figure 3). We performed a thyrohyoidopexy using polypropylene 2.0 (five stitches wrapped around the hyoid bone and through the thyroid lamina submucosally). The remaining left-side part of the wound was closed on the left side using polyglactin 910 2.0 (Ethicon Company, New Jersey, USA). After completing the repair, hemostasis was ensured, and two Jackson-Pratt drains were inserted into the para-esophageal groove. The wound was closed in layers, and the skin was approximated using staples. After being tracheostomized and connected to a ventilator, the patient was transferred to the intensive care unit. Where he stayed for two days before being transferred to a ward. He remained in the hospital for a total of 30 days, during which time he was primarily under the care and supervision of the psychiatric team.

## Discussion

**Figure 4:**
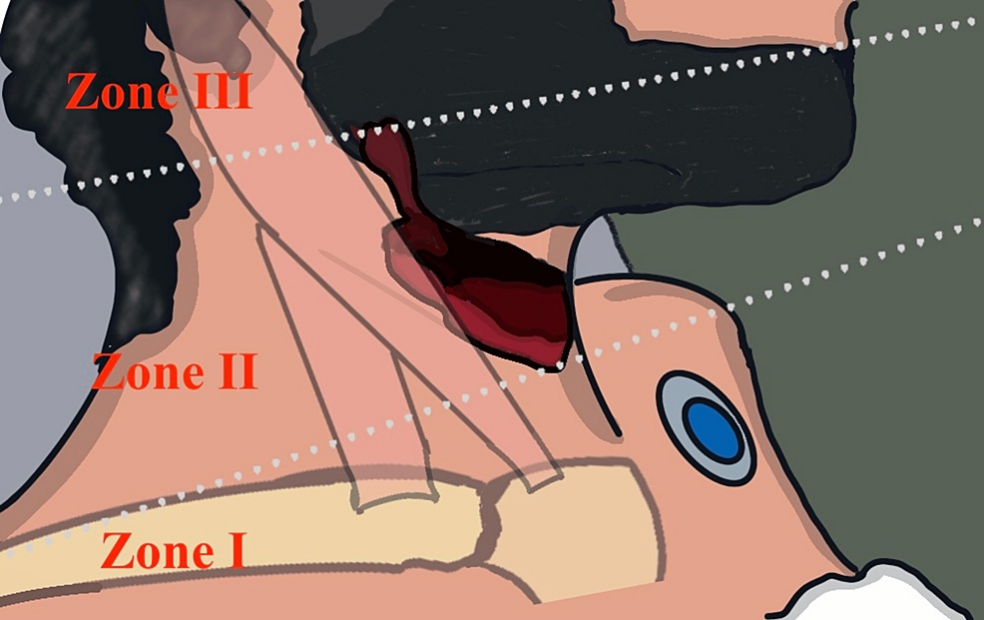
An illustration that demonstrates the neck zones relation to the location and extension of the neck injury.

Nearly 5%-10% of all trauma cases presented to the ED are directed to the head and neck [1,3]. PNIs can be split into three zones based on the anatomical level of the damage [1,4] (Figure 4).

Zone I reaches the level of the cricoid cartilage between the clavicles and sternal notch. Zone II is the area between the cricoid and the inferior mandibular border [1,3]. The area in which this case has the injury and that we want to highlight has the greatest concentration of critical structures, including the jugular veins, larynx, hypopharynx, and cranial nerves X, XI, and XII, as well as the common carotid artery and internal and external carotid arteries [3-5]. Zone III is the region between the mandibular angle and the skull base [4,6]. Historically, an early neck exploration was necessary for all cases, regardless of the location [7].

This invasive intervention was in favor of early detection of vascular bleeding that could be missed by physical examination [8]. This resulted in a high rate of negative exploration within this entity [3,8], which subsequently contributed to increased mortality by delaying management and increasing the rate of complications and length of stay [7]. Classifying the neck into zones was introduced in 1980 by Biffl et al. Biffl et al. demonstrated that targeted care of PNIs was safe and efficient in an 18-year prospective study [9]. Penetrating vascular injuries require careful diagnosis and management for a complex entity with a multifaceted nature. The study demonstrated that "hard signs" of vascular injury in stable patients mandate immediate intervention if the injury is located within zone II [7,9]. Such signs include external bleeding, internal bleeding into the esophagus, trachea, or mouth, signs of a pulsatile or expanding hematoma in the neck, acute neurological symptoms, tracheal deviation, or elevation of the floor of the mouth from a hematoma. However, the anatomical accessibility of zones I and III is limited, which requires a more selective evaluation approach [7,8]. To effectively manage vascular injuries, surgeons must employ a range of diagnostic modalities [8]. Additional evaluation, including angiography, bronchoscopy, and esophagoscopy, may be used to assess the extent of injury and guide treatment decisions [7]. Such evaluations can be intrusive and uncomfortable for the patient and carry significant risks of complications [3].

Nowadays, most surgeons support treating penetrating wounds selectively based on the neck zone, the patient’s physiological condition, and the clinical findings after evaluating the entire neck as a single entity [3]. This implies the chosen operative management of patients based on thorough physical examinations and targeted diagnostic investigations for middle-zone neck injuries [3,7,8]. Nevertheless, there is agreement on the need for urgent exploration in cases with concomitant esophageal damage, progressing mediastinal or subcutaneous emphysema, pneumothorax, and extremely acute dyspnea that necessitates intubation [2,10,11]. Although less than 1% of traumatic damage involves direct trauma to the airway, making it a highly uncommon injury, these injuries can substantially impact the overall prognosis [11,12]. Due to the weakness of the connective tissues in zone II and the high exposure of this location [2], this is the most common area subjected to impact, accounting for 50%-80% of all trauma affecting the neck [10,12]. In our case, the injury was at the level of the hypopharynx below the cricoid level; the fracture of the right thyroidal alar cartilage alongside the left thyroid alar cartilage damage was indicative of the severity of the impact resulting from the knife. This impact substantially resulted in compromising the airways of our patient. Excluding other potentially life-threatening airway injuries is crucial. Subcutaneous emphysema could indicate pneumopericardium or pneumoperitoneum; although unusual, it can be fatal [13]. Fortunately, our patient did not have such a manifestation. 

Upon the primary survey, we ensured that the airway was protected; the literature does not agree on a single airway intervention for patients with airway compromise [7]. Treatment options discussed range between open surgical intervention with or without tracheotomies and simple medical management under observation [2,7]. The treatment option chosen depends on the aerodigestive tract status [7]. The location and severity of the injury are also determining factors [11]. In cases of laryngotracheal injuries, as in this patient, we initially insert the ET tube into the trachea as a temporary measure. Choosing size 7 mm was based on the patient’s age and build. This patient was a thin man with a laryngeal injury. Otherwise, we would have gone with the size of 8 mm [14]. The airways should receive special attention because bleeding within the small neck compartments may appear stable from the outside but can gradually compromise the airway and eventually completely obstruct it. That justified shifting this patient to the OR, intending to establish a definitive airway (tracheostomy). Tracheostomy was achieved after accomplishing good control of bleeding from zone II structures. The patient's proximal and distal ends of the transected anterior jugular veins were ligated. Upon exploration was done after airways were maintained. Repair should be started only after maintaining an adequate airway [11,15].

Prior to commencing the repair procedure, the patient's cervical region was subjected to a thorough re-examination to detect any comminuted fractures. Given its technical ease relative to wire fixation, we first utilized it to stabilize the minimally displaced fractures when the perichondrium was still in place. Various techniques for fixing the laryngeal framework have been reported. Including laryngeal framework fixing techniques using nonabsorbable monofilament sutures, stainless steel wire, and titanium mini plates [15,16]. Polypropylene is a permanent monofilament with the benefit of high tensile strength and helps with stronger perichondrium and cartilage approximation, but it also has the drawback of granuloma formation when exposed intraluminally. Another type mentioned in the literature is polydioxanone, which is significantly less reactive and a good option in the event of intraluminal exposure. Prior to the laryngofissure closure, a nylon suture is used to hold the stent in place by passing it through the neck, larynx, and stent. A couple of 2-0 nonabsorbable monofilament sutures were used to close the laryngofissure [15]. After closure, a flexible esophagoscopy was performed for evaluation.

The drains are removed, when output becomes negligible, and there are no signs of subcutaneous collections. In most patients undergoing transcervical repair of laryngeal fractures, swallowing function will be reduced in the early postoperative term. Until a modified barium swallow demonstrates a functional, secure swallow, nasogastric feeding should be continued. Due to the patient's background of psychological instability and the tendency to frequently removing the NGT tube, stent removal was not performed before the nasogastric feeding [15], as that would make it difficult to keep the tube in place.

## Conclusions

This report at hand discusses a rare yet oftentimes fatal presentation of penetrating neck trauma, which poses a significant risk of immediate life-threatening events due to the potential damage to the airway resulting from the presence of a multitude of vital structures in the neck region. The risk of immediate life-threatening events makes the initial assessment and management of the affected individual of utmost importance. The Advanced Trauma Life Support guideline emphasizes the need to manage the airway as a crucial part of the primary survey, followed by a meticulous evaluation of the patient's vital signs and hard signs upon presentation. The use of an endotracheal tube may be necessary until the definitive airway is established, maintaining the airway and preserving the blood supply during the repair process. Additionally, the selection of the appropriate type of suture is essential for each repair, considering the location and severity of the injury.
